# Pulmonary nocardiosis in a patient with pemphigus foliaceus: case report and literature review

**DOI:** 10.1186/s12879-020-05673-5

**Published:** 2021-01-06

**Authors:** Ning Luo, Shifan Tan, Xiaocai Li, Si Liu, Shivank Singh, Mafeng Chen, Weiye Yang, Yanhong He, Chunna Chen, Min Liang

**Affiliations:** 1 Department of Respiratory and Critical Care Medicine, Maoming People’s Hospital, MaoNanQu, WeiMing Road 101 Hao, Maoming City, Guangdong China; 2 Southern Medical University, Guangzhou, China; 3 Department of Otorhinolaryngology, Maoming People’s Hospital, Maoming, China; 4 Department of Laboratory Medicine, Maoming People’s Hospital, Maoming, China; 5 Department of Scientific Research, Maoming People’s Hospital, Maoming, China; 6 Department of Dermatology, Maoming People’s Hospital, Maoming, China

**Keywords:** Nocardia asteroides, Pemphigus foliaceus, Diabetes, Corticosteroids, Case report

## Abstract

**Background:**

Nocardiosis is an uncommon opportunistic infection seen in immunocompromised patients or those with a dysfunctional immune system. Nocardia asteroides infection in patients with Pemphigus foliaceus (PF) has never been reported.

**Case presentation:**

We report an interesting case of nocardiosis-characterized by pulmonary intra-cavitary infection, in a 54-year-old man with PF and diabetes mellitus. The man finally recovered from the infection.

**Conclusions:**

This is the first case reporting pulmonary nocardiosis in a patient with PF. We recommend that physicians be aware of nocardiosis in patients with pemphigus as a possible cause of underlying infectious disease to avoid misdiagnoses and mismanagement.

## Background

Pemphigus is a chronic, potentially lethal disorder of the skin and mucous membranes. Long-term therapy with corticosteroids to control disease might, unfortunately, cause generalized immuno-suppression. We present a rare case, in which immuno-supression was further potentiated by diabetes mellitus, resulting in an opportunistic pulmonary infection with Nocardia asteroides.

## Case presentation

This case report has been approved by the ethics committee of Maoming People’s Hospital.

**Fig. 1:**
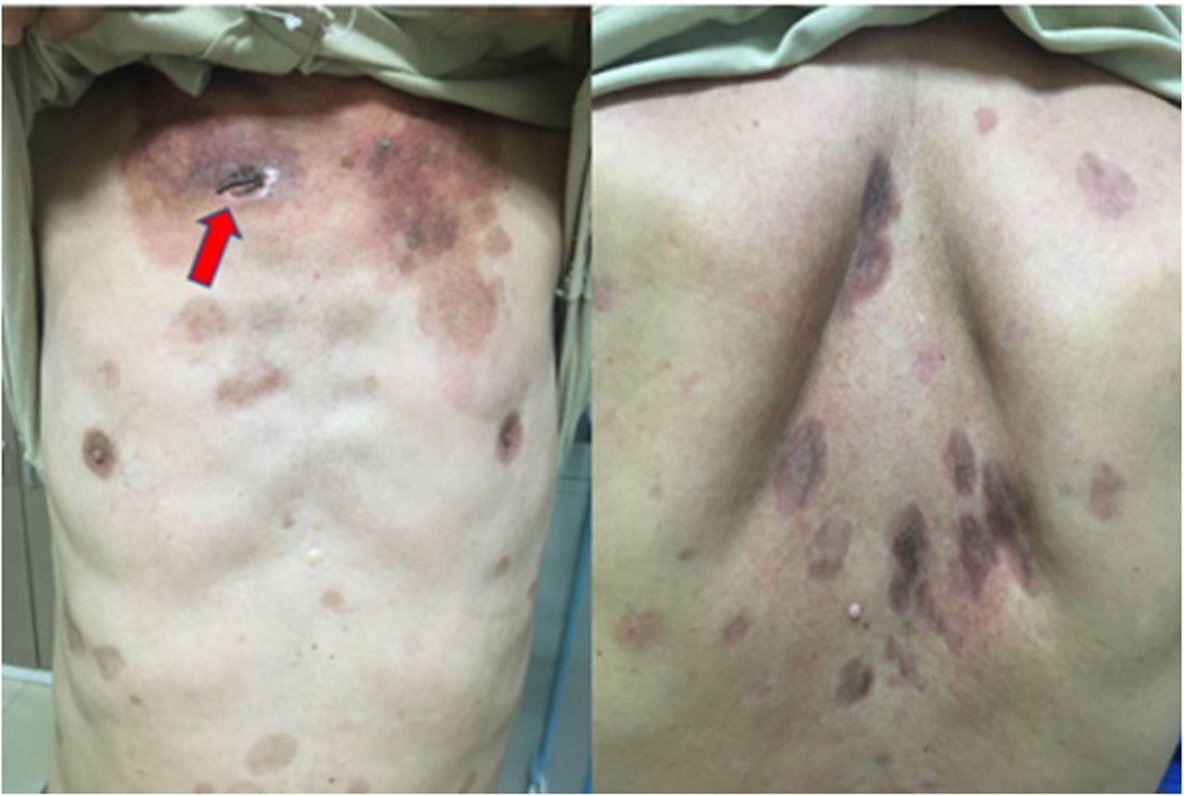
Multiple, crusted lesions of PF on the anterior chest and upper back (the scar is marked with a red arrow)

**Fig. 2:**
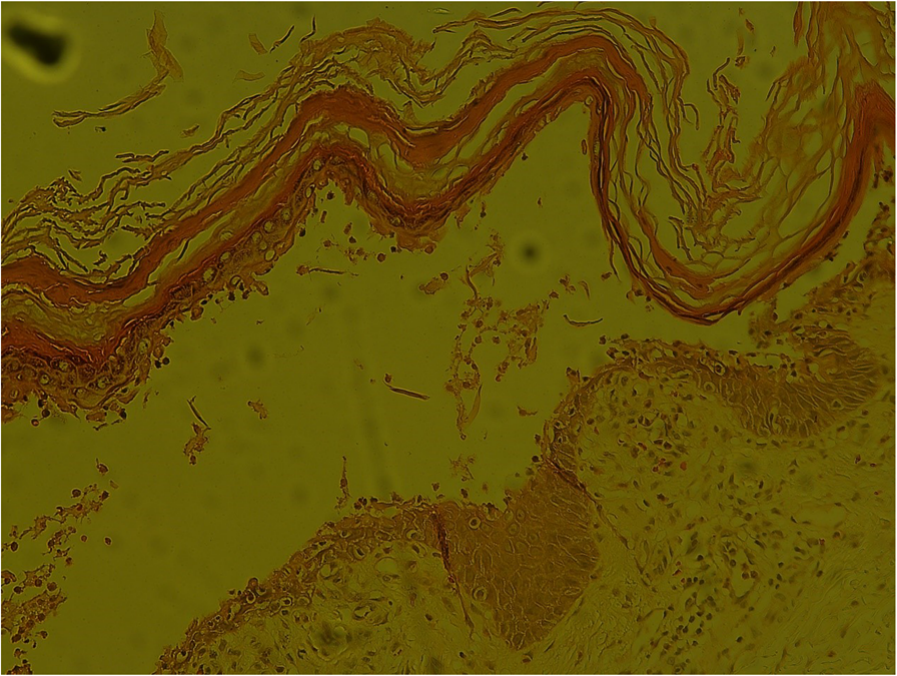
Punch biopsy from the anterior chest revealed decreased intercellular adhesions and acantholysis (Hematoxylin & Eosin, × 200)

**Fig. 3:**
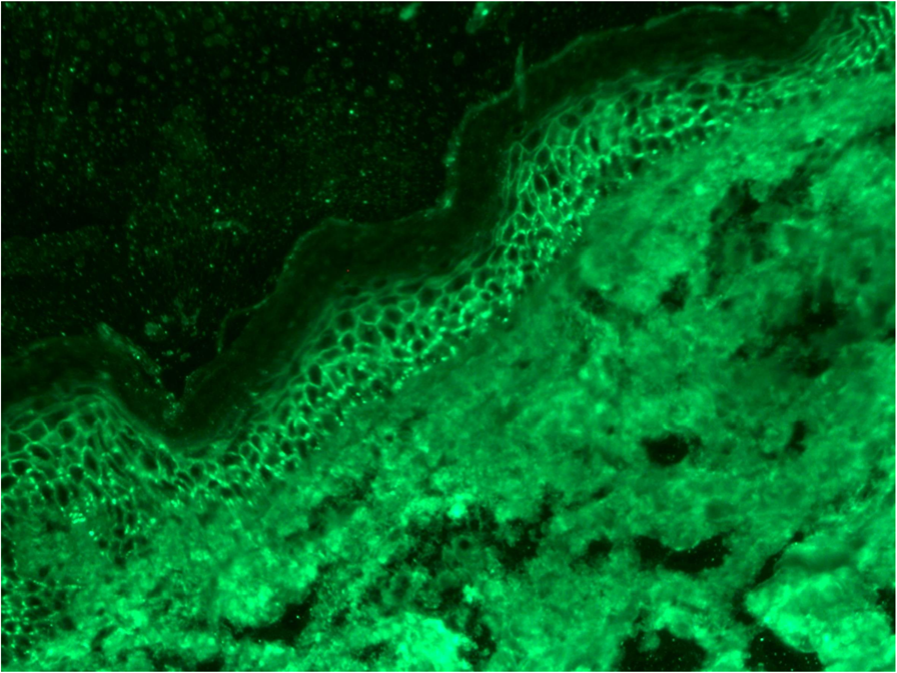
Direct immunofluorescence: Intercellular immune IgG deposits present in the epidermis

**Fig. 4:**
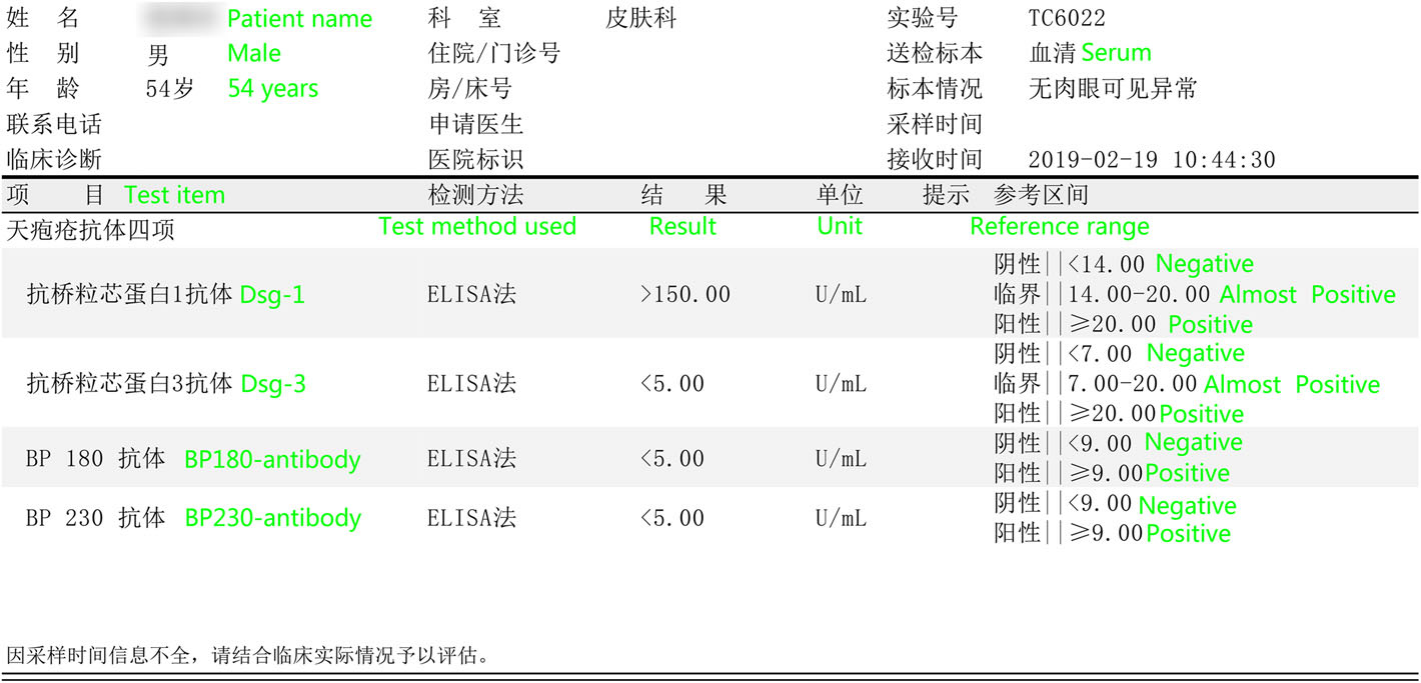
Results of serological findings (ELISA method). Dsg-1 antibody with a level more than 150 U/mL, Dsg-3 antibody was within the normal range

A 54-year-old man with diabetes mellitus was admitted to Maoming People’s Hospital on 26, April 2020. He was diagnosed with PF ten months prior to admission. The diagnosis was based on clinical manifestations and laboratory findings [1], 1) Localized superficial blisters with erosion and crusts appeared on the area of upper trunk (Fig. 1.) 2) Punch biopsy from anterior chest revealed decreased intercellular adhesions and acantholysis (Fig. 2.) 3) Direct immunofluorescence showed intercellular IgG deposition in the epidermis (Fig. 3.) 4) Detection of serum anti-desmoglein-1 (Dsg-1) antibody with a level more than 150 U/mL (ELISA method) whereas the serum anti-desmoglein-3 (Dsg-3) antibody was within the normal range (Fig. 4.).He was initially prescribed daily oral prednisone(60 mg) for PF and achieved complete remission. After 3 months of maintenance therapy, the dose of prednisone was gradually and carefully tapered to 20 mg P.O. daily. The patient was also diagnosed with diabetes mellitus 1 year ago, but did not follow medical advice for management. Consequentially, he had poorly-controlled blood glucose levels. He worked as a farmer and had a 10 pack-year smoking history.

Three months before admission the patient was wounded by a tree branch on the anterior chest but didn’t seek medical service at the time. He began feeling fatigued about a month later which was followed gradually by a productive cough and right-sided chest pain. The patient presented to the local clinic with a sudden high grade fever(39.6 °C), chills, sputum evolution and shortness of breath, where he was thought to have bacterial pneumonia and received oral and intravenous antibiotic therapy. However, he failed to respond to the therapy and was transferred to Maoming People’s Hospital.

**Fig. 5:**
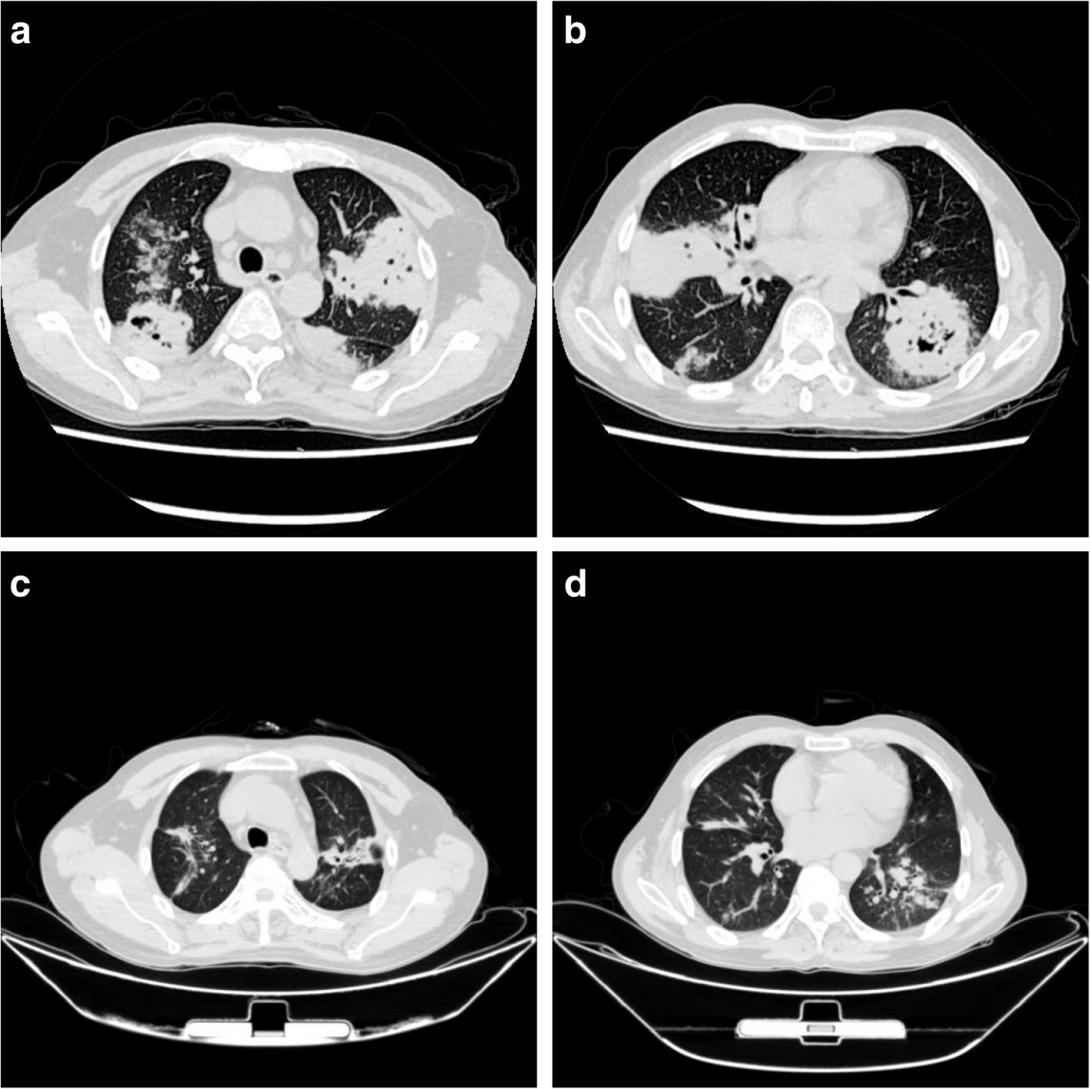
CT comparison at hospital admission (**a** and **b**) and 3 months post hopital-discharge (**c** and **d**); On admission: Thick-walled cavities in multiple lobes of both lungs (**a**: upper lobes, **b**: Middle lobe and lower lobes); **c** and **d**: lesions improved following treatment for 3 month

**Fig. 6:**
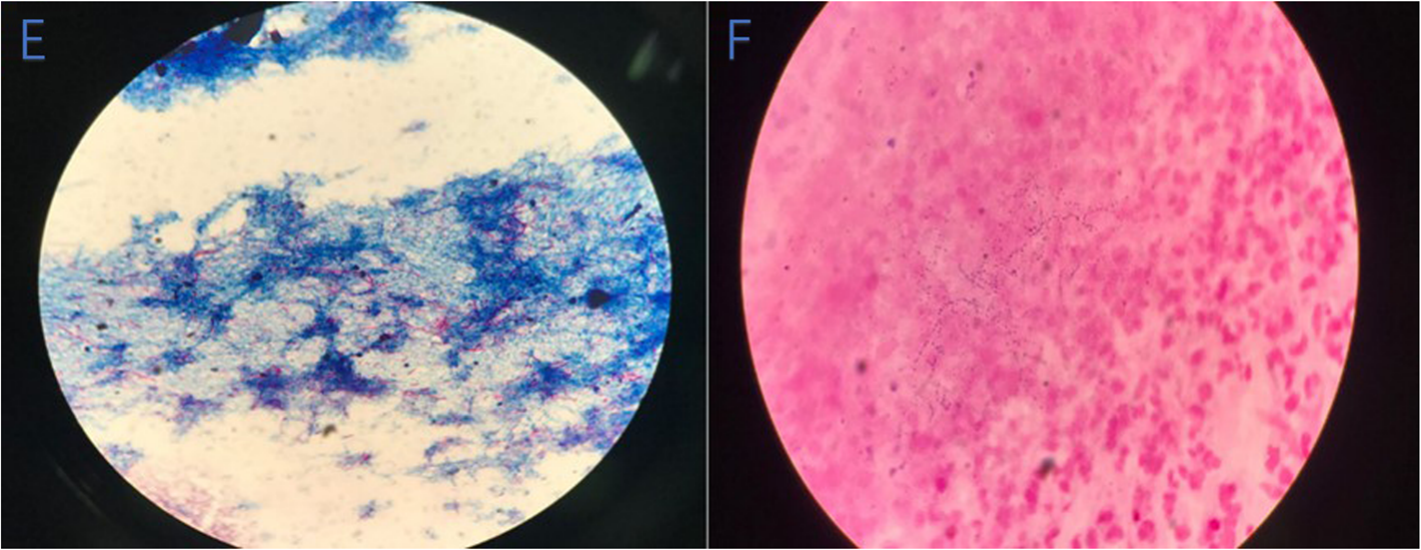
Results of sputum culture. E: Branching, thin filamentous acid-fast bacteria on modified (1%) ZN stain, F: Gram-positive, filamentous, delicate branching rods with an irregular staining pattern

During primary evaluation, the patient exhibited a poor general condition and strong breathlessness. Initial vital signs were: Body temperature: 39.4 °C; Heart rate: 116 bpm; Respiratory rate: 26 breaths/min; Blood pressure: 101/60; Oxygen saturation: 75%. Physical examination revealed multiple skin lesions on the chest, shoulder, and upper back. An old scar was visible on the right sternal border- on the same area as the skin lesions (Fig. 1.). Lung auscultation revealed end-inspiratory rales in both lung bases. Blood tests showed a WBC count of 12,910 cells/ul, Rapid C-Reactive Protein count of over 200 mg/L and Hemoglobin count of 11.7 g/dl. His glycated hemoglobin (HbA1c) was 10.10%.Human immunodeficiency serology was negative. A chest CT was performed and revealed several ‘fungal ball’ like inclusions in thick-walled cavities present in multiple lobes of both lungs (Fig. 5.).Blood culture, Tuberculin test, Xpert MTB/RIF test, serum (1,3)-β-D-glucan (BDG) and serum galactomannan (GM) antigen detection test returned negative. However, specimen from Broncho-alveolar Lavage fluid (BAL) and multiple sputum cultures revealed *Nocardia spp*. (Fig. 6.). The organism was later identified as Nocardia brasiliensis by rRNA gene sequencing. Results of other complementary studies to detect extra-pulmonary organ involvement were negative.

A combination of intravenous cefotaxime (2.25 g,Q8h), amikacin (0.4 g/day) and oral trimethoprim-sulfamethoxazole (0.32 g trimethoprim, 1.6 g sulfamethoxazole) daily were initiated after diagnosing Nocardia asteroides, along with continuation of prednisone (20 mg/day) for pemphigus. Insulin was also administered to manage blood glucose levels.

Seven days post-admission, his body temperature became normal and we was told by the patient that the symptoms of cough and breathlessness were markedly improved as well. Blood tests showed WBC counts and levels of Rapid C-Reactive protein within the normal range. Following another 7 days of sequential therapy, the patient was discharged with instructions to continue therapy with oral trimethoprim-sulfamethoxazole for next 3 months.

After discharge, the patient did well. His pemphigus was completely under control with a daily oral dose of 20 mg prednisone and progressive improvements took place in cough and breathlessness up to the last visit on August 1, 2020. A follow-up CT was performed in the meanwhile, which revealed further improvement from nocardia pneumonia (Fig. 5.).

## Discussion and conclusion

Pemphigus is a rare but serious chronic blistering disorder of the skin and mucous membranes [2]. It is usually categorized into two distinct forms, Pemphigus vulgaris (PV) and Pemphigus foliaceus (PF), each with its own variants(e.g. The endemic form of PF, also known as Fogo selvagem (FS)) [3–5]. Over the past decades, rare forms of pemphigus have been described, including paraneoplastic pemphigus, IgA pemphigus, and pemphigus herpetiformis, etc. [5]. Even though the disease has a chronic clinical course, the risk of morbidity and mortality is considerable. Before the advent of corticosteroids, estimated mortality rate at the end of 5 years was approximately 100% [6]. With the availability of corticosteroids, the mortality rate has declined to 5–10% [7].

Bacterial infection with *Staphylococcus aureus, Proteus vulgaris and Pseudomonas aeruginosa,* is the most common complication after long-term administration of corticosteroids in pemphigus patients. However, pulmonary nocardiosis in a patient with pemphigus has been rarely reported in recent cases.

Pulmonary nocardiosis is an opportunistic infection, which usually occurs in immunocompromised patients, alcoholics, and patients on long term immunosuppressive therapy [8]. It has a variable presentation on Chest CT, usually mimicking tuberculosis, fungal balls, or lung cancer [9]. Isolation and identification of *Nocardia spp*. from cultures of sputum, BAL, and biopsy helps establish the diagnosis [10]. A combination of sulfonamides, amikacin, and ceftriaxone is recommended as first-line therapy [11]. In our patient’s case, the long-term management of pemphigus with oral prednisone and poor diabetic control, together, increased the patient’s immunodeficiency, finally resulting in pulmonary infection with nocardia. The most probable route of infection is indeed a point of debate- we speculate that apart from the possibility of inhalation, the patient’s history of chest trauma should be taken into consideration as well, since pulmonary nocardiosis can potentially result from exposure to bacteria via abraded skin [12].

A literature search based on PubMed/MEDLINE was conducted until July 10, 2020. A total of nine accessible cases reporting pemphigus patients with nocardiosis were retrieved (Table 1) [13–18]. One of the studies reported four cases of pulmonary nocardiosis but the presented clinical data was insufficient. The overall gender composition of studies was 3 females / 6 males. All cases were PV patients who had undergone corticosteroid treatment. Varying doses of sulfonamide antibiotics, particularly trimethoprim-sulfamethoxazole, was the most used therapeutic regimen. One patient with severely deteriorated health died within a short period of time after biopsy confirmation of nocardiosis, others improved or got cured of pulmonary nocardiosis after therapy. We noticed that except for the study with insufficient data, patients in most case-reports (4/5) had a history of tapered dose of corticosteroids in pemphigus management before presenting with and being diagnosed with nocardiosis. This brings us to a very concerning thought: was it possible that the reduced dosage of corticosteroids during the management of pemphigus resulted in immunological modifications and subsequently increased the risk of contracting opportunistic infections such as nocardiosis? If so, caution should be exercised while deciding optimal dosage as well as the time period for corticosteroid adjustment. Apart from the single patient who died right after diagnosis of nocardia, all others had a good treatment outcome. Therefore, early diagnosis along with timely and effective therapy is extremely important for the management of nocardiosis.

**Table 1:** Summary of cases reporting nocardiosis in patients with pemphigus

Study	Age/gender	Comorbidity	Clinical presentation	Radiological features	Specimen	Nocardia spp.	Antibiotic therapy	Tapering dose of corticosteroids before admission	Outcome
Asilian2006 [13]	56y/man	Chronic bronchitis	Skin abscess, cough and dyspnea	CT: Segmental opacities in the middle zone of the right lung with loculated right pleural effusion	Pleural fluid and sample of skin abscesses	UA	Cotrimoxazole:50 mg/kg/day, i.v	Yes	Improved
Murdock1990 [14]	50y/woman	Diabetes mellitus	Sore throat, cough,chest pain,fever, chills	CXR: A large cavitary lesion and an infiltrate in the right upper lung	Sputum	Nocardia brasiliensis	Trimethoprim-sulfamethoxazole: ten days during hospitalization, then minocycline, 100 mg twice a day for 4 months after discharged	No	Improved
Rahdar2020 [15]	40y/man	UA	UA	UA	BAL, DNA sequencing	N. cyriacigeorgica	Trimethoprim-sulfamethoxazole: 15 mg/kg/day	UA	Cured
Rahdar2020 [15]	41y/man	UA	UA	UA	DNA sequencing	N. cyriacigeorgica	Trimethoprim-sulfamethoxazole: 15 mg/kg/day	UA	Cured
Rahdar2020 [15]	39y/man	UA	UA	UA	DNA sequencing	N. otitidiscaviarum	Trimethoprim-sulfamethoxazole: 15 mg/kg/day	UA	Cured
Rahdar2020 [15]	58y/man	UA	UA	UA	BAL, DNA sequencing	N. cerradoensis	Trimethoprim-sulfamethoxazole: 15 mg/kg/day	UA	Cured
Miller1967 [16]	UA/woman	UA	Lethargy and malaise	CXR: A left suprahilar mass and pleural effusion	Pleural fluid	UA	Sulfisoxazole:8 mg daily	Yes	Improved
Martin2000 [17]	58y/man	Hypertension	Cough,fever and low level of consciousness	Diffuse cerebral lesions	Lung and brain biopsies	UA	Cefuroxime and Ciprofloxacin	UA	Died
Chosidow1990 [18]	57y/woman	Breast malignancy, rheumatoid arthritis and diabetes mellitus	Fever, shivers generalwasting and skin abscess	CXR: A middle-lobe nodular density CT: Hypodensity of the third segment of the liver	Skin biopsy and blood cultures	UA	Trimethoprim-sulfamethoxazole: 400 and 2400 mg/day, Amikacin: 500 mg/day	Yes	Improved
Present case	54y/man	Diabetes mellitus	Fever,breathlessness,chest pain	CT: Thick-walled cavities existed in multi-lobes of both lungs	Sputum,BAL, RNA sequencing	Nocardia brasiliensis	Trimethoprim-sulfamethoxazole, intravenous Cefotaxime and Amikacin	Yes	Improved

*UA* Unavailable, *BAL* Bronchoalveolar lavage fluid, *CT* Computerized tomography, *CXR* Chest X-Ray, *PV* Pemphigus vulgaris, *PF* Pemphigus foliaceus

According to our extensive searches, this is the first case to be reported of pulmonary nocardiosis in a patient with concomitant PF. Combining the findings of the patient in this case-report and other related reports, we recommend that physicians be aware of nocardiosis in patients with pemphigus as a possible cause of underlying infectious disease to avoid misdiagnoses and mismanagement.

## Data Availability

The datasets used and/or analysed during the current study are available from the corresponding author on request.

## Abbreviations

PF: Pemphigus foliaceus
FS: Fogo selvage
PV: Pemphigus vulgaris
Dsg-1: Anti-desmoglein-1
Dsg-3: Anti-desmoglein-3
UA: Unavailable
BAL: Bronchoalveolar lavage fluid
CT: Computerized tomography
CXR: Chest X-Ray
